# On Simple Chancres of the Preputial Margin

**Published:** 1882-07

**Authors:** I. E. Atkinson


### SELECTIONS.

---

### ON SIMPLE CHANCRES OF THE PREPUTIAL MARGIN.

---

In giving a title to the brief article that follows, I have avoided using the term "chancroid" for the reason that, apart from any question of unity or duality of origin of venereal ulcers, it is misleading and incorrect. Clerc, who coined the word, designated by it the result of inoculation with the virus of the infecting or syphilitic chancre upon an individual who has, or who has had, constitutional syphilis. He also held that this ulcer might be inoculated upon healthy persons, producing only local effects, and that, occasionally, from it might spring the infecting chancre and constitutional syphilis. This ulcer, considering it the analogue of varioloid, he called "chancroid." (Clerc, "Memoire du chancröide syphilitique," Paris, 1854). It is needless to say that no American writer attaches such a meaning to the term "chancroid" at the present time. Then, again, in the sense employed by Bumstead and others (indeed, nearly every one in this country), "chancroid" is not recognized by European authors (if we except some in Great Britain) as designating the "simple chancre."

Since, then, the term is not in general use in the greater portion of the medical world, and since it has been given, by nearly all those who employ it in America, a signification different from that it originally had with its author and those most familiar with his opinions, there is no good reason why it should not be discarded. In speaking of the "chancroid," therefore, if we use the term "simple chancre," it may be easily distinguished from the "infecting chancre," and at the same time we employ language everywhere intelligible and not likely to be distorted in its meaning.

The characters of simple chancre have been often and ably described, and are for the most part easy of recognition. Every writer recognizes the fact, however, that it sometimes becomes difficult, even impossible, to ascertain the real nature of a venereal ulcer from its physical characters alone ; time alone can afford a solution to the enigma. The difficulties attending the formation of a diagnosis seem, in some measure, to depend upon the locality of the ulcer, more than upon other influences.

Considering for a moment the features by which we determine the nature of a given chancre, features presented by the ulcer itself, without reference to the various accessory phenomena and historical data that so greatly aid in reaching conclusions, we observe that a simple chancre rarely exists alone, that it deeply excavates the tissues upon which it is implanted, that it secretes an abundant pus from an uneven, grayish, "worm-eaten" floor, presents to the touch a base without induration, or slightly resistant from simple inflammatory infiltration, often extends by ulceration, and multiplies by contagious infection of the neighboring parts. By these and other features the simple chancre may usually be recognized, but it is certainly true that the diagnosis often requires far more deliberation than most authors would lead us to believe. The difficulty will depend not only upon the simulation of other forms of ulcer by the simple chancre, but also upon the resemblance to the simple chancre sometimes offered by the infecting chancre. The former may, indeed, acquire a toughness and induration from various kinds and degrees of irritation, which may usually be distinguished, but which occasionally defy the powers of the most experienced observers, especially where, as frequently occurs, the injudicious application of irritating and feebly caustic remedies induces a cartilage-like density, the true nature of which, time and the abandonment of the treatment alone can determine. On the other hand, the infecting chancre may present characters indistinguishable from those of simple chancre, either from the obscurity of its proper signs, or from their absence.

It is not my purpose to enter upon this tempting field at this time. For the present, I desire to speak of some of the peculiarities presented by simple chancres upon and just within the free border of the prepuce. Although statistics of this subject are rather scanty and unavailable (all simple chancres of the prepuce usually being considered together), there can be no doubt that this region is frequently attacked. Le Fort (Jullien, "Malad. Vener.," p. 320) reports, of six hundred and fifteen cases of simple chancre, seventy-eight where the free border of the prepuce was the part affected.

In the conditions about to be described, the sores are developed only in those persons in whom the glans penis is habitually covered with a redundant prepuce, or in whom congenital phimosis is present. At first the ulcer does not differ from simple chancre in any other locality. If primary, the usual pustule may initiate the series of changes; or this may not be observed, either on account of its ephemeral duration, or because the moisture of the parts has prevented its formation. If secondary to ulcers elsewhere, the primary lesion is usually seated upon the preputial mucous membrane or upon the glans. If, then, one is fortunate enough to see the lesion in its earlier stages, it will be found to present the characters of ordinary simple chancres; or, in the case of phimosis, if it is out of sight, it will reveal its presence by pain, soreness, redness, and œdema, and by purulent or sanguineo-purulent discharge. It may happen that the chancre will go through its phases without departing from the type of its kind. More commonly, there are observed changes that may be, with propriety, attributed to peculiarities of position, and which may be briefly described.

The simple chancre, more active and more inflammatory than the infecting or syphilitic chancre, situated upon the loose meshwork of connective tissue forming the prepuce, rapidly occasions an amount of inflammatory exudation that thickens the parts and destroys their natural elasticity. Where the prepuce is by nature redundant and prolonged beyond the extremity of the glans penis, the sore, primarily marginal, or secondary to more deeply seated chancres, exciting these changes, often rapidly induces a traumatic phimosis in those cases where phimosis is not of congenital origin. This may be more or less complete. There results, obstruction to the free escape of the copious purulent discharge of the chancre, and the epithelial lining of the prepuce, bathed in this fluid, becomes softened, macerated, and is often stripped off, exposing the subjacent parts to the dangers of auto-inoculation. These chancres rarely remain single, and when situated upon the preputial margin often present an aspect to which Bumstead and Taylor have directed attention ("Venereal Diseases," 1879, p. 375). They are often "*exulcerous*, or superficial, their floor being nearly or quite on a level with the surrounding integument a fact which has been attributed to the constant irritation to which they are subjected from the subpreputial discharge and the urine." Locality, however, exerts here influences that produce other somewhat peculiar results.

The patient, either alarmed at the processes going on out of sight, or in his endeavors to practise the necessary care for insuring cleanliness and cure, attempts to retract the now stiff and narrowed or completely phimosed circle of the prepuce, over the glans penis. In these manipulations the weakened epithelial lining yields and cracks, just as constantly occurs in analogous conditions in other parts of the body. These cracks or fissures, running parallel to the axis of the penis, become inoculated with the chancrous virus, and there are produced a number of simple chancres arranged around the free rim of the prepuce, preserving, for the most part, the linear arrangement, but possessing all the properties of the chancre from which they sprung. There are now present one or more subpreputial simple chancres, together with an indefinite number along the preputial ring, all secreting freely, and accompanied by a greater or less degree of inflammation. Up to this point, while the processes have been interesting, and possessing peculiarities depending upon their location, there have been no complications preventing ready diagnosis and prognosis. When, however, the conditions above described have lasted for a number of days, the œdema and inflammatory exudation bring about alterations offering some obscurity. That portion of the prepuce nearest the ulcers begins to become indurated.

Induration of simple, non-infecting chancres is not an uncommon occurrence, notwithstanding many prevalent statements to the contrary. It must be understood, however, that this induration is usually of a purely inflammatory character, and may nearly always be distinguished by the skilled observer from the peculiar hardness characteristic of the infecting chancre. Nevertheless, the fact remains that the purely inflammatory infiltration of the simple chancre may become intense, while the specific induration of the infecting chancre frequently is very insignificant; so that uncertainty and error are often not only excusable, but may even be unavoidable. Indeed, were even expert observers obliged in all cases to form their diagnosis from the absence or presence of induration alone, less would be said and written about the ease with which one may decide upon the nature of venereal ulcers. Every one is familiar with the cartilage-like mass of induration often excited in a simple chancre by the foolish attempt of its bearer to treat himself, under the aspiring supervision of some "experienced" non-professional friend, when the crystal of cupric sulphate or the stick of lunar caustic or some other irritating application, are employed with a faith and perseverance worthy of a better cause.

In simple chancres of the preputial margin, the inflammatory infiltration of which we have been speaking, can produce an induration hardly less exactly resembling the specific induration of infecting chancre. It cannot be said, however, that the induration in these cases is identical with that dense, gristly hardness that characterizes the most pronounced forms of infecting chancres; it rather resembles the less decided induration that one meets in the common run of these. It is quite dense, quite unyielding, but gives to the touch a sense of *fibrous* resistance, and is not sharply circumscribed, though it involves more or less regularly the whole preputial ring. This condition is not assumed until after the lapse of a week or two at least, previous to which the diagnosis may usually be made readily. Probably the most instructive opportunity of realizing the difference between the cartilaginous hardness of the most pronounced infective character and simple inflammatory induration is afforded in cases of specifically indurated, subpreputial infecting chancre, where the irritation of the latter has induced the infiltration of which we are speaking, in the tissue of the redundant prepuce.

It often becomes impossible to decide upon the nature of a chancrous infiltration of the prepuce from a consideration of the physical characters of the sore alone. The patient, under the circumstances we have been considering, will present a reddened, swollen, and infiltrated prepuce, from the phimosed orifice of which a more or less copious, creamy, or sero or sanguineo-purulent fluid escapes. Slight retraction of the prepuce, all that can be practised, displays the encircling ulcers. To the touch the tissue serving as a basis for these chancres is indurated and resistant. To decide upon the exact nature of the process, we may be compelled to have recourse to other signs. It is seldom that the patient can be confronted with the author of his malady. The date of the contagious exposure often cannot be ascertained. For our guidance there remain the condition of the inguinal glands and the results of auto-inoculation. Fortunately we here meet guides that generally serve to lead us out of our perplexing indecision. The inguinal glands will either remain unaffected or will undergo simple irritative or virulent inflammation, such as characterizes the course of simple chancres. These glands, however, may fail to supply quite satisfactory evidence. In certain individuals, scrofulous for the most part, they, in common with the lymphatic glands generally, are enlarged, resistant to the touch, and while lacking the stony hardness of the painless inguinal glands of true syphilitic infection, may, at times, give rise to mistakes, especially in view of the pre-existing anomalous condition of the preputial lesions.

The results obtained from auto-inoculation often lend, just here, valuable assistance ; for while it can no longer be doubted that, to a certain extent, infecting chancres are auto-inoculable, they are so to a slight degree, not to be compared with the readiness with which the virus of the simple chancre excites ulcerative action.

Usually no special danger attends the course of simple chancres situated and behaving as above described. Their duration is apt to be longer, both from the difficulty of applying proper remedies and the liability to almost indefinite reproduction by contagion. Unless extensive ulceration exists far back upon the prepuce or upon the glands, very destructive action is not to be apprehended, nor does there seem to be any special tendency toward phagedena. There is often a considerable amount of suffering from the acute inflammation present, and the passage of urine is sometimes painful. The phimosis that is produced often remains permanently, after recovery, from scar formation ; and firm indurations are apt to persist long after the disease has been cured. Weeks usually elaspe before recovery is established.

The treatment of these chancres differs from that ordinarily pursued only from peculiarities of situation. Circumcision, or the various operations for phimosis, are often practised, and are sometimes really necessary. The dangers of subsequent inoculation of the cut surfaces are very great, sometimes insuperable, and, unless there is imperative need to set free the imprisoned glans penis, on account of the intensity of the destructive process, cases will be found to do well, usually, without it. It may be considered advisable to operate, when from the degree of inflammation, or the appearance of phagedena, destruction to the tissues is imminent. It is but seldom that caustics should be used in the treatment of these chancres. In the great preponderance of cases they do more harm than good. A caustic, to be used efficiently, *must* destroy all of the chancrous virus ; failing to do this, as it must obviously fail in nearly all of these ulcers, on account of their situation, it becomes an agent of mischief.

When phimosis is present, the patient should be warned against attempts to uncover the glans ; the parts behind the preputial ring should be thoroughly cleansed with warm water, thrown in through an appropriate syringe. As soon as all discharges have been washed out, some mildly astringent solution should be injected under the foreskin. Weak solution of zinc sulphate, of ferric potassio-tartrate, of aromatic wine, will be found to answer admirably. To the ulcers themselves care must be taken to avoid too stimulating treatment. Any of the various washes usually beneficial in the treatment of these ulcers may be employed. It will often be found advantageous to separate the surfaces of the prepuce and glans by pledgets of lint or of soft linen or cotton fabric soaked in the wash or applied dry. Iodoform often exerts the same charming influence here as in other localities. It should be applied in powder. Nothing but its abominable odor, which persists in spite of many ingenious devices to conceal it, prevents its general adoption. Calomel powder often does much good, as do also bland absorbent powders.

Under any treatment the duration of simple chancres of the preputial margin is somewhat more protracted than is usual for those of other situations. The phimosis often resulting from the cicatrization of the chancres can only be relieved by an operation.—*Dr. I. E. Atkinson in Med. News.*

